# Complete Genome Sequence of *Lactobacillus rhamnosus* Strain LRB

**DOI:** 10.1128/genomeA.01208-16

**Published:** 2016-11-03

**Authors:** Saswati Biswas, Indranil Biswas

**Affiliations:** Department of Microbiology, Molecular Genetics and Immunology, University of Kansas Medical Center, Kansas City, Kansas, USA

## Abstract

*Lactobacillus rhamnosus* is a Gram-positive facultative heterofermentative lactic acid bacterium. It is often isolated from the gastrointestinal tract, mouth, vagina, and fermented dairy products. We have isolated the *L. rhamnosus* strain LRB from a healthy baby tooth that had naturally fallen out. Here, we report the annotated whole-genome sequence of LRB.

## GENOME ANNOUNCEMENT

*Lactobacillus rhamnosus* is a Gram-positive lactic acid bacterium isolated from diverse ecological niches, including the gastrointestinal tract, oral cavity, vagina, and fermented dairy products. The majority of the strains of this species are considered to be probiotics, especially for their roles in the intestine and vagina. We have isolated *L. rhamnosus* LRB from a baby tooth that had fallen out naturally. The complete genome sequence of this strain was determined using the PacBio long-read sequencing method and *de novo* assembly (1). Sequencing data were generated with 16.11-fold coverage and assembled using the assembly program SMRT2.3.0_HGAP3_May132016 (2).

The genome of *L. rhamnosus* LRB consists of a circular chromosome of 2,934,954 bp with 46.78% G+C content. The whole-genome sequence was annotated with the NCBI Prokaryotic Genome Annotation Pipeline (PGAP) (3). This genome contains 2,749 total genes, among which 2,672 are total protein-coding sequences (2,428 coding genes and 244 pseudogenes). This strain contains 15 rRNA genes, 59 tRNA genes, and one clustered regularly interspaced short palindromic repeat (CRISPR) array. This isolate did not contain any plasmids. A total of 11 base modification motifs were found in the LRB genome by PacBio single-molecule real-time (SMRT) sequencing.

The most extensively studied strain, *L. rhamnosus* GG (ATCC 53103), a gut isolate, consists of a genome of 3,010,111 bp (GenBank accession no. NC_013198) (4). Therefore, the LRB genome is 75,157 bp shorter than that of GG. BAGEL3 analysis shows that *L. rhamnosus* strains contain a varied number of predicted bacteriocin loci, generally ranging from two to four loci. Like GG, the strain LRB contains four predicted bacteriocin loci (5). The LRB genome contains the pilus gene cluster *spaFED*, along with a class C sortase in the locus. However, LRB lacks the *spaCBA* gene cluster of GG and is not expected to produce functional pili (6). This difference may indicate why the respective strains reside in different habitats.

In a phylogenetic context, the Rapid Annotations using Subsystems Technology (RAST) server predicted *L. rhamnosus* strain HN001 (accession no. NZ_ABWJ00000000.1), a food isolate, to be the closest neighbor of LRB (7). However, the genome sequence of HN001 used for this prediction was a draft genome (8). The second and third closest neighbors are intestinal isolates LMS2-1 and GG, respectively. It is not unusual that the strains isolated from different ecological niches have a close phylogenetic relationship. Intestinal isolates, LMS2-1, E800, and ATCC 21052, are shown to individually occupy the closest branch of a phylogenetic tree with a food isolate (9). This fact indicates that these intestinal isolates may have first originated from food and eventually evolved. Since LRB has a slightly larger genome (20,546 bp) than the food isolate and closest neighbor, *L. rhamnosus* HN001 (10), we can speculate that LRB has also originated from food and evolved by acquiring a small number of genes to become an oral resident. According to a recent report, *L. rhamnosus* is capable of adapting to a new environmental niche with little genetic variation (9).

### Accession number(s).

This complete genome sequence has been deposited in GenBank under the accession no. CP016823.
